# *CTLA-4 *+49A/G and CT60 gene polymorphisms in primary Sjögren syndrome

**DOI:** 10.1186/ar2136

**Published:** 2007-03-06

**Authors:** Jacques-Eric Gottenberg, Pascale Loiseau, Mariam Azarian, Chun Chen, Nicolas Cagnard, Eric Hachulla, Xavier Puechal, Jean Sibilia, Dominique Charron, Xavier Mariette, Corinne Miceli-Richard

**Affiliations:** 1 Rhumatologie, Institut Pour la Santé et la Recherche Médicale (INSERM) U802, Université Paris-Sud 11, Hôpital Bicêtre, 78 rue du Général Leclerc, Assistance Publique-Hôpitaux de Paris (AP-HP), 94275 Le Kremlin Bicêtre, France; 2 INSERM 396, Immunologie et Histocompatibilité, Hôpital Saint-Louis, 1 avenue Claude-Vellefaux, AP-HP, 75475 Paris cedex 10, France; 3 Institut Cochin, Unité de biologie moléculaire, Hôpital Cochin, 27 rue du Faubourg Saint-Jacques, 75679 Paris cedex 14, France; 4 Médecine Interne, Hôpital Claude Huriez, 2 avenue Oscar Lambret, 59000 Lille, France; 5 Rhumatologie, Hôpital du Mans, 194 avenue Rubillard, 72037 Le Mans, France; 6 Rhumatologie, Hôpital Hautepierre, 1 avenue Molière, 67098 Strasbourg, France

## Abstract

*CTLA-4 *encodes cytotoxic T lymphocyte-associated antigen-4, a cell-surface molecule providing a negative signal for T-cell activation. *CTLA-4 *gene polymorphisms have been widely studied in connection with genetic susceptibility to various autoimmune diseases, but studies have led to contradictory results in different populations. This case-control study sought to investigate whether *CTLA-4 *CT60 and/or +49A/G polymorphisms were involved in the genetic predisposition to primary Sjögren syndrome (pSS). We analysed *CTLA-4 *CT60 and +49A/G polymorphisms in a first cohort of 142 patients with pSS (cohort 1) and 241 controls, all of Caucasian origin. A replication study was performed on a second cohort of 139 patients with pSS (cohort 2). In cohort 1, the *CTLA-4 *+49A/G*A allele was found on 73% of chromosomes in patients with pSS, compared with 66% in controls (*p *= 0.036; odds ratio (OR) 1.41, 95% confidence interval (CI) 1.02 to 1.95). No difference in *CTLA-4 *CT60 allelic or genotypic distribution was observed between patients (*n *= 142) and controls (*n *= 241). In the replication cohort, the *CTLA-4 *+49A/G*A allele was found on 62% of chromosomes in patients with pSS, compared with 66% in controls (*p *= 0.30; OR 0.85, 95% CI 0.63 to 1.16). Thus, the *CTLA-4 *+49A/G*A allele excess among patients from cohort 1 was counterbalanced by its under-representation in cohort 2. When the results from the patients in both cohorts were pooled (*n *= 281), there was no difference in *CTLA-4 *+49A/G allelic or genotypic distribution in comparison with controls. Our results demonstrate a lack of association between *CTLA-4 *CT60 or +49A/G polymorphisms and pSS. Premature conclusions might have been made if a replication study had not been performed. These results illustrate the importance of case-control studies performed on a large number of patients. In fact, sampling bias may account for some contradictory results previously reported for *CTLA-4 *association studies in autoimmune diseases.

## Introduction

Polymorphisms in *CTLA-4*, the gene encoding cytotoxic T lymphocyte-associated antigen-4, have been widely studied in connection with genetic susceptibility to various autoimmune diseases [1], but studies have led to contradictory results in different populations.

Among *CTLA-4 *gene polymorphisms, a G to A transition at position 49 (+49A/G) of exon 1 leads to an alanine to threonine amino acid substitution at codon 17 in the leader peptide (A17T), and a C to T transition at position 60 (CT60) is located within the 3'-untranslated region [2]. The G allele of +49A/G has been associated with a predisposition to many autoimmune diseases (reviewed in [1]). Both polymorphisms are in linkage disequilibrium, which warrants haplotype analysis in studies of *CTLA-4 *polymorphisms. The CT60 G allele has been reported to increase susceptibility to several autoimmune diseases [2], and a functional approach provided evidence for lower mRNA levels associated with the CT60 G allele [2].

Downie-Doyle and colleagues have recently reported a significant association of the *CTLA-4 *+49A/G*A allele and of the *CTLA-4 *+49A/G*A allele carrier haplotypes with primary Sjögren syndrome (pSS), especially in patients with anti-SSA or anti-SSB antibodies, in a study including 111 Australian patients with pSS and 156 controls [3].

The aim of our study was to investigate in a large case-control study whether *CTLA-4 *CT60 and/or +49A/G SNPs were involved in genetic predisposition to pSS in French patients.

## Materials and methods

### Patients

A first cohort of 142 unrelated patients with pSS diagnosed in accordance with the European American consensus group criteria [4] (37% without autoantibodies, 30% with anti-SSA antibodies only, and 33% with both anti-SSA and anti-SSB antibodies) and 241 healthy blood donors were genotyped for *CTLA-4 *CT60 and +49A/G polymorphisms. A second independent cohort of 139 patients with pSS was further genotyped for *CTLA-4 *+49A/G polymorphisms in a replication study. The geographical origin and the clinico-biological characteristics of the patients in this second cohort were the same as those in the first. In this second cohort, 27% of patients were anti-SSA and anti-SSB negative, 35% had anti-SSA only and 38% had both anti-SSA and anti-SSB. All patients and controls were Caucasians and provided informed consent.

### Genotyping

After the isolation of genomic DNA from peripheral blood mononuclear cells, *CTLA-4 *CT60 and +49A/G polymorphisms were genotyped by restriction fragment length polymorphism with the use of *Bbv*I (+49A/G) and *Nla*III (CT60).

### Statistical analysis

Allelic and genotypic frequencies of *CTLA-4 *CT60 and +49A/G polymorphisms were compared between patients and controls by using a two-sided χ^2 ^test. All genotyped SNPs were in Hardy–Weinberg equilibrium. *CTLA-4 *(+49A/G or CT60) haplotypes, constructed with the PHASE program, were also examined for association with pSS. *P *< 0.05 was considered significant.

## Results

In the first cohort of patients with pSS, the A allele of the *CTLA-4 *+49A/G polymorphism was found on 73% of chromosomes in patients with pSS, in comparison with 66% in controls (*p *= 0.036, odds ratio (OR) 1.41, 95% confidence interval (CI) 1.02 to 1.95; Table 1). No significant difference in *CTLA-4 *+49A/G*A allele frequencies was observed among subgroups of patients according to their anti-SSB and/or anti-SSA status (Table 1). No difference in *CTLA-4 *CT60 allelic or genotypic distribution was observed between patients (*n *= 142) and controls (*n *= 241). *CTLA-4 *(+49A/G or CT60) haplotype distribution mirrored the *CTLA-4 *+49A/G*A allele excess among patients with pSS (A/A 48%, A/G 26%, G/G 26%, G/A 0.4%; in comparison with A/A 45%, A/G 21%, G/G 34% among controls), leading to an excess of +49A/G*A allele carrier haplotypes among patients (*p *= 0.03, OR 1.41, 95% CI 1.02 to 1.95).

**Table 1:** Allellic frequencies of *CTLA-4 *49A/G polymorphism among patient controls

*CTLA-4*+49A/G	Allele frequencies						*p*	Odds ratio (95% CI)
			
	pSS	SSA+ and SSB+	SSA+ only	Ac+	Ac0	Controls (*n *= 241)		
Cohort 1	*n *= 142	*n *= 47	*n *= 43	*n *= 90	*n *= 52			pSS vs controls
A (Thr)	208 (73)	68 (72)	61 (71)	129 (72)	79 (76)	318 (66)	0.036	1.41 (1.02–1.95)
G (Ala)	76 (27)	26 (28)	25 (29)	51 (28)	25 (24)	164 (34)	0.036	0.70 (0.51–0.98)
Cohort 2	*n *= 139	*n *= 52	*n *= 49	*n *= 101	*n *= 38	Controls (*n *= 241)		
A (Thr)	173 (62)	59 (57)	66 (67)	125 (62)	48 (63)	318 (66)	NS	0.85 (0.62–1.15)
G (Ala)	105 (38)	45 (43)	32 (33)	77 (38)	28 (37)	164 (34)	NS	1.17 (0.86–1.60)
Total	*n *= 281	*n *= 99	*n *= 92	*n *= 191	*n *= 90	Controls (*n *= 241)		
A (Thr)	381 (68)	127 (64)	127 (69)	254 (66)	127 (71)	318 (66)	NS	1.08 (0.84–1.40)
G (Ala)	181 (32)	71 (36)	57 (31)	128 (34)	53 (29)	164 (34)	NS	0.92 (0.71–1.19)

Numbers in parentheses are percentages. pSS, primary Sjögren syndrome; Ac+, presence of anti SSB and/or anti-SSA; Ac0, absence of anti-SSA or anti-SSB antibody; CI, confidence interval; NS, not significant.

To avoid the possibility of a false positive association of *CTLA-4 *+49A/G*A with pSS as a result of the somewhat small sample size of our first cohort, and because the *CTLA-4 *+49A/G*A allele has been only marginally associated with autoimmune diseases compared with the *CTLA-4 *+49A/G*G allele [1], we performed a replication study on a second independent cohort of 139 patients with pSS. In this second cohort, the *CTLA-4 *+49A/G*A allele was found on 62% of chromosomes in patients with pSS, compared with 66% in controls (*p *= 0.30; OR 0.85, 95% CI 0.63 to 1.16; Table 1). Thus, the *CTLA-4 *+49A/G*A allele excess among patients with pSS from the first cohort was counterbalanced by its under-representation in the second cohort. When the results from the patients in both cohorts were pooled (*n *= 281), there was no difference in *CTLA-4 *+49A/G polymorphism allelic or genotypic distribution in comparison with controls (*p *= 0.53, OR 1.09, 95% CI 0.84 to 1.4; Table 1). The sex ratios among patients (0.97) and controls (0.06) were different. We therefore investigated *CTLA-4 *+49A/G polymorphism genotypic distribution among males and females in the control group and found that it was not statistically different (*p *= 0.1), thus excluding any possible gender effect.

Our results therefore demonstrate a lack of association between *CTLA-4 *CT60 or +49A/G polymorphisms and pSS among Caucasians.

## Discussion

The results from our first cohort were very close to those from the study of Downie-Doyle and colleagues [3], with a significant association of pSS with the +49A/G*A allele and with the +49A/G*A allele carriers haplotypes. The association observed in the first cohort, of two haplotypes bearing the same allele (*CTLA-4 *+49A/G*A), was actually more probably due to the statistical weight of the *CTLA-4 *+49A/G*A allele than to a true functional effect of two different haplotypes, bearing either *CTLA-4 *CT60*C or *CTLA-4 *CT60*T alleles, each having opposite functional effects on *CTLA-4 *mRNA expression [2].

In fact, our results suggest a false positive association of *CTLA-4 *+49A/G*A allele with pSS in the first cohort of patients. When data were pooled (cohorts 1 and 2), no significant association was found with the *CTLA-4 *+49A/G polymorphism in our Caucasian population of patients with pSS. This was not the consequence of different origin or different clinico-biological characteristics of the patients from the two cohorts and could only be the result of a sampling bias. Indeed, the findings observed in our first cohort of patients, as those from Downie-Doyle and colleagues [3], were unexpected because there are only rare examples of association of the *CTLA-4*A/G*A allele with autoimmune diseases [5-7]. Consequently, we might have made premature conclusions if a replication study had not been performed.

## Conclusion

Our study illustrates the necessity to include a large number of patients in genetic case-control studies. In fact, sampling bias may partly account for some contradictory results previously reported for *CTLA-4 *association studies in autoimmune diseases.

## Abbreviations

CI = confidence interval; CTLA-4 = cytotoxic T lymphocyte-associated antigen-4; OR = odds ratio; pSS = primary Sjögren syndrome; SNP = single nucleotide polymorphism.

## Competing interests

The authors declare that they have no competing interests.

## Authors' contributions

JEG contributed to the study design, performed the statistical analysis and drafted the manuscript. PL and DC supervised genotyping and contributed to DNA samples collection. MA and CC performed genotyping. NC performed PHASE analyses. EH, XP, and JS contributed to DNA samples collection. XM and CMR supervised the study design and gave valuable advice to JEG and PL. All authors read and approved the final manuscript.
